# The Effects of Thymoquinone on Viability, and Anti-apoptotic Factors (BCL-XL, BCL-2, MCL-1) in Prostate Cancer (PC3) Cells: An In Vitro and Computer-Simulated Environment Study

**DOI:** 10.15171/apb.2019.058

**Published:** 2019-08-01

**Authors:** Javad Saffari_Chaleshtori, Ehsan Heidari-Sureshjani, Fahimeh Moradi, Esfandiar Heidarian

**Affiliations:** ^1^Student Research Committee, Shiraz University of Medical Sciences, Shiraz, Iran.; ^2^Young Researchers and Elites Club, Islamic Azad University, Shahrekord Branch, Shahrekord, Iran.; ^3^Cellular & Molecular, Research Center, Basic Health Sciences Institute, Shahrekord University of Medical Sciences, Shahrekord, Iran.; ^4^Clinical Biochemistry Research Center, Basic Health Sciences Institute, Shahrekord University of Medical Sciences, Shahrekord, Iran.

**Keywords:** Thymoquinone, Apoptosis, Cancer, Simulation

## Abstract

***Purpose:*** Since active plant ingredients can induce apoptosis in many tumors, in this study we evaluate the apoptotic effects of thymoquinone (TQ) on PC3 cells. Also, we predicted the interaction of TQ with BCL-XL, BCL-2, and MCL-1anti-apoptotic factors by computer-simulated environment.

***Methods:*** PC3 cells were treated with different concentrations of TQ (0- 80 µM) and IC_50_ was determined using 3-(4, 5-dimethylthiaztol-2-yl)-2, 5-diphenyltetrazolium bromide (MTT) assay. Apoptotic and cytotoxicity effects of TQ were analyzed using flowcytometry and comet assay, respectively. Changes in energy and the molecular interactions of TQ with BCL-XL, BCL-2 and MCL-1 anti-apoptotic factors were investigated using simulation.

***Results:*** IC_50_ value was 40 µM. TQ led to the destruction of the genome of PC3 cells and inducing apoptosis. Molecular dynamics (MD) revealed that the root mean-square deviation (RMSD), root mean square fluctuation (RMSF), radius of gyration (Rg), and the number of hydrogen and hydrophobic bonds between TQ and residues of BCL-2, BCL-XL and MCL-1were significantly (*P*<0.001) changed. TQ makes a more stable and stronger connection with BCL-XL compared to BCL-2 and MCL-1 and inhibits BCL-XL non-competitively.

***Conclusion:*** Our results demonstrated that TQ not only led to apoptosis, at least partly, due to reduction in the Coil, Turn, and Bend structure of BCL-XL but also caused a decrease in the Rg and RMSD value of BCL-XL, MCL-1, and BCL-2.

## Introduction

Nowadays, cancer is one of the most important concerns worldwide and has persuaded many scientists to perform extensive studies to know more about the mechanism underlying its development and also the approaches to prevent and cure the disease.^1,2^ Prostate cancer (PC) is the most prevalent cancer in men and is the second leading cause of death due to cancer after lung cancer in males.^3^ PC is initially dependent on androgens for proliferation, but eventually progresses to be androgen-independent phenotype PC in the androgen-independent phenotype becomes highly resistant to chemotherapy in advanced stages of the disease.^4^Androgen ablation therapy is mainly used for the treatment of hormone-sensitive PC. However, in most men usually after 2 to 3 years, metastatic PC leads to an androgen-independent state.^5^ Many of the natural plant ingredients play an important role in cancer prevention.^6^ These natural compounds such as alkaloids, terpenes, lignans and flavonoids in various plants have anti-tumor properties.^7^ Thymoquinone (2-isopropyl-5-methyl-1,4-benzoquinone) (TQ) is a bioactive plant ingredient which is abundantly found in plants such as *Nigella sativa* Linn.^8^ TQ has been reported to have antioxidant,^9^ anti-inflammatory,^10^ anti-neoplastic^11^ and anti-poisoning activities in malignancies such as PC,^12^ osteosarcoma,^13^ fibro sarcoma^14^ and myeloblastic leukemia.^15^ This compound has a strong antioxidant effect by which it prevents tumor cell growth in many cancers.^16^

Inhibition of cancer cell growth is often achieved by the activation of apoptotic regulatory factors. BCL-2, MCL-1and BCL-XL have been identified as three important factors in this pathway.^17^ BCL-2 family plays a key role in regulation and inhibition of apoptosis and increase in its activity gives rise to a wide spectrum of cancers.^18^ Reduction in BCL-2 gene expression is the hallmark of the beginning of apoptosis which reinforces the effects of anti-tumor therapy. Over expression of BCL-2 causes resistance to anti-cancer therapy. BCL-2 interacts with a subset of pre-apoptotic molecules including BAD, BAX, BIM, BAK, BID, and BIK directly.^19^ Also, BCL-XL and MCL-1, as anti-apoptotic factors, play a critical role in the inhibition of apoptosis.^20^ Therefore, the aim of this study was to determine the genotoxic effects of TQ on PC3 cell line as well as predicting interactional molecular dynamic (MD) mechanisms of TQ with BCL-XL, BCL-2, and MCL-1 anti-apoptotic factors by computer-simulated environment based on molecular docking software (Auto Dock, Gromacs and VMD).

## Materials and Methods

The human PC cell line, PC3, was purchased from Pasteur Institute of Iran (Tehran, Iran). TQ was obtained from Santa Cruz Biotechnology Inc. (Santa Cruz, CA, USA). RPMI 1640 medium and fetus bovine serum (FBS) were prepared from Gibco (Rockville, MD, USA). Pen/Strep antibiotics were purchased from Abcam (San Francisco, CA, USA). 3-(4,5-dimethylthiaztol-2-yl)-2,5-diphenyltetrazolium bromide (MTT) was purchased from Sigma-Aldrich (St. Louis, MO). All other chemicals used were of analytical grade.

### Experimental Methods

#### Cell culture

PC3 cell line was cultured in RPMI1640 medium supplemented with 10%heat-inactivated FBS, penicillin (100 U/mL) and streptomycin (100 U/mL). The flasks were kept in a humidified incubator containing 5% CO_2_ at 37°C.^21^

### Cell viability

The viability of cells against cytotoxic effects of TQ was performed by MTT colorimetric assay. Briefly, 5×10^3^cells were cultured in a 96-well plate containing RPMI 1640 phenol-red free medium, supplemented with 10% FBS. After overnight incubation, cells were treated with different concentrations of TQ (0- 80 µM) for 48 h in a humidified 5% CO2 incubator.^21^ Then, cells were treated with 12mM MTT solution and were incubated for 4 h. Optical density was measured at 490 nm with 670 nm as the reference wavelength by a micro plate reader (Stat Fax-2100, Spain). Each test was carried out in triplicate. Viability percentage was calculated as follows: (OD value of treated group/OD value of control group) ×100 and non-treated cell viability was set as 100%.^22^

### Alkaline electrophoresis and comet assay

Based on IC_50_ value_,_ 20 and 30 µM of TQ concentrations (two concentrations lower than IC_50_) were used in alkaline electrophoresis. PC3 untreated cells were used as negative control and positive control was treated with 50µM H_2_O_2_.^23^ Slides were cleaned by methanol and heated gently. A 300 µL of 1% agarose dissolved in PBS was poured on slides and after inserting lamella, the slides were put on ice packs to harden the first agarose layer. Slides were incubated at 37°C for 24 h, after removing lamella. 10 µL of prepared cells was mixed with 80 µL of 1 % low melting point agarose in PBS. Then, 10 µL of the solution was poured over the first layer of agarose and the lamella was immediately inserted to make the middle layer. The third layer of agarose was made using 1% agarose in PBS. Slides were incubated for 24hin lysis buffer containing 100 mM Na_2_ EDTA, 10% DMSO and 2.5 mM NaCl, pH 10, and 1% triton X-100. In the next step, slides exposed to alkaline buffer solution containing 1 mM Na_2_EDTA, 0.3 M NaCl and pH=13 for 30 minutes. The slides were subjected to electrophoresis (25V, 300 mA, and 40 min). Then, slides were rinsed three times using neutralizing solution comprising 0.4M Tris HCl (pH 7.5). Slides were fixed in 95% ethanol three times, stained in 20 µg/mL ethidium bromide and finally analyzed using fluorescent microscope with 40X magnification (Olympuse BX51, Tokyo, Japan). One hundred cell images were analyzed for each concentration using CASP v.1.2.2 software.^24^

### Flowcytometry

PC3 cells were cultured in 6-well plates at a density of 1 × 10^4^/well and were treated with three different concentrations of TQ (20, 30, and 40 µM). After treating PC3 cells with TQ for 48h, cells were harvested and washed twice with PBS. Then, the PC3 cells were mixed with binding buffer and incubated with an annexin-V/PI double staining solution at room temperature for 15 minutes according to the manufacture’s protocol. Then, the 12 000 stained cells were analyzed by flow cytometry and the percentage of apoptotic cells was calculated.

### Simulation and Molecular Dynamics (MD) of proteins

Protein structure of anti-apoptotic proteins including BCL-2, BCL-XL, and MCL-1 were obtained from the RCSB protein data bank with PDB ID: 2W3L, 2YXJ, and 3KZ0, respectively. PubChem compound databank (from National Center for Biotechnology Information) was used to obtain the structure of TQ with CID: 10281. For docking simulations, by applying the automated docking tool (AutoDock), the hetero atoms were removed and H-atoms were added to the protein structures.^25^ MD simulation of BCL-2, BCL-XL, and MCL-1 in absence of TQ was done by Gromacs software v. 4.5.4.

### Molecular docking

Docking simulation studies were conducted on three anti-apoptotic proteins and TQ using Lamarckian genetic algorithm (LGA) method^26^ with automated docking tool (AutoDock 4.2.3 version). For each protein, a virtual network with x,y,z dimensions was created, though the size of the box has been changed depending on the TQ size. AutoDock grids were calculated for regularly spaced points at intervals of 0.375 A° contained within a cube based on the BCL-2, BCL-XL, and MCL-1. Other docking parameters were left at the software’s default values set. LGA was selected to specify the best conformations in 200 independent trials of each TQ. Also, the LigPlot plus v.2.1 software was used to describe protein–ligand interactions between the TQ and amino acids.

### MD simulation of protein-ligand complexes

MD simulation of BCL-2, BCL-XL, and MCL-1 docked with TQ was ran by Gromacs v.4.5.4 software as described previously.^27^ The temperature was set to 300 K for all the simulation times. In this study, docking and dynamic simulations were performed using bit system with Intel^®^Core^TM^ i7 CPU Server.

### Statistical analysis

Flowcytometry analysis was performed with GraphPad Prism 5 software. All the data were analyzed using Statistical Package for the Social Sciences software, version 22 (SPSS, Inc., Chicago, IL, USA). Inhibitory concentration of 50% (IC_50_) was calculated by the Probit procedure. The alkaline comet assay parameters were calculated by one-way ANOVA followed by Tukey post hoc test. Paired-sample *t* test was used for MD analysis. *P* value less than 0.001 was considered significant.

## Results and Discussion

Today, extensive studies have been carried out to reduce the mortality and morbidity of cancer diseases. During recent years, plant drugs and their derived compounds have attracted the attention of many scientists.^28^ Some studies have revealed that some of these compounds are able to induce or inhibit apoptosis in tumor cells.^29^

**Figure 1:**
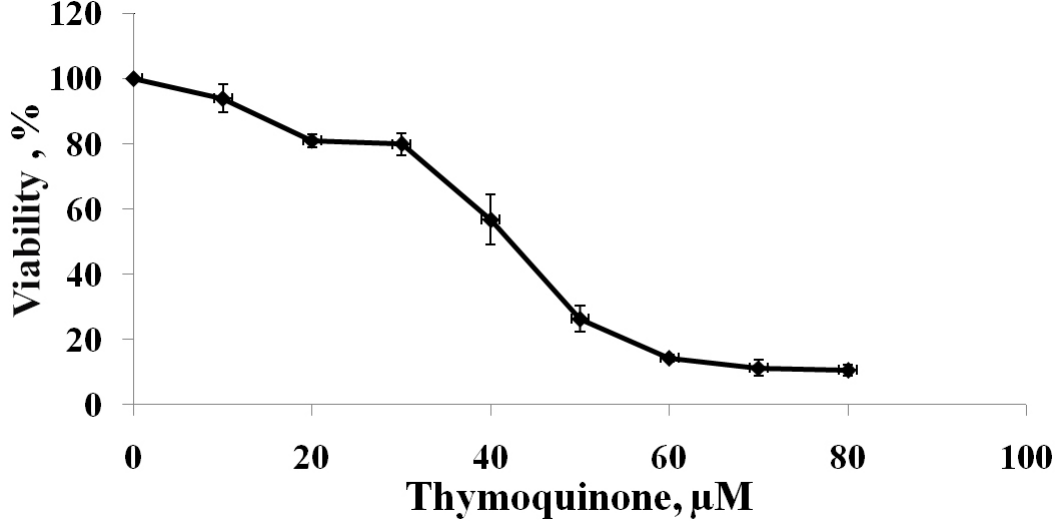
Inhibitory effects of TQ on PC3 cells viability. The viability of PC3 cells was measured by MTT assay for 48h at various concentrations of TQ. The data are expressed as mean ± SD three independent observations.

Our findings showed that TQ can induce apoptosis strongly in PC3 cells. Figure 1 shows the cytotoxicity effects of TQ on PC3 cells. TQ declined the cell viability percentage gradually with the elevation of TQ concentration after 48 h. In our study, the IC_50_ value of TQ was 40µM which is in line with previous study.^30^ It is reported that TQ can induce a significant up-regulation of reactive oxygen species (ROS) expression and free radicals in PC3 cells which lead to catalyze oxidation of lipids and oxidize cysteine residues in proteins accompanied by altering protein structure and function. These changes due to ROS may lead to growth inhibition.^31^ Therefore, the reduction in PC3 cells viability in our study can result from, at least partly, ROS expression and free radicals.

**Figure 2:**
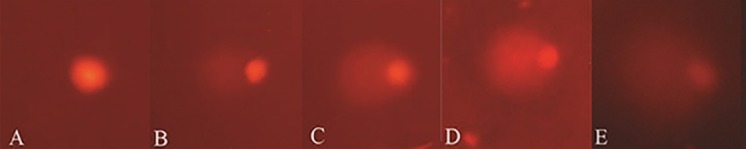
Genotoxicity effects of TQ on PC3 cells. (A) Negative control cells (without any tail), (B-D) cells treated with 20, 30, 40 µM concentrations of TQ respectively, € Cells treated with H_2_O_2._

In the present study, the effects of TQ at different concentrations on DNA fragmentation of PC3 cells were surveyed by comet assay (Table 1,Figure 2). The tail/head ratios of DNA were elevated by a dose dependent manner of TQ and maximized at IC_50_concentration (40 µM) for negative control and treated cells. The ratio of the tail to head diameter on image D was higher, compared to other images. The tail/head cell ratio diameter is regarded as an appropriate criterion to determine the intensity of breakage. The ratios less than 10% displayed minor damage while higher ratios were considered as an indicator of severe or major damage. In our study, positive control (cells treated with 50 µM H_2_O_2_) had the longest tail which implied severe damage of the genome (Table 1). In the present study, the concentrations higher than IC_50_ led to cell death and determination of genome breakage was not assessed by comet assay. Also, our results show that the concentration of 40 µM has inflicted the most damage on PC3 cancer cells (Table 1).

**Table 1:** Genotoxicity Effects of TQ on PC3 Cells

**Parameter**	**n**	**Control (A)**	**20 µM (B)**	**30 µM (C)**	**40 µM (D)**	**H**_2_ **O**_2_ **50 µM (E)**
Head DNA	100	43.3±0.7	8.2±0.2^a^	3.4±0.3^ab^	4.6±0.5^ab^	6.6±0.5^ac^
Tail DNA	100	0.8±0.1	2.0±0.1^a^	4.3±0.1^ab^	10.2±1.3^abc^	15.0±1.1^abc^
Head DNA%	100	98.2±0.1	79.7±0.7^a^	40.4±1.0^ab^	33.5±0.3^abc^	32.8±0.5^abc^
Tail DNA%	100	1.8±0.1	20.3±0.7^a^	59.6±1.0^ab^	66.5±0.3^abc^	67.2±0.5^abc^

A, B, C, and D are treated groups with 0 (negative control), 20, 30, and 40 µM of TQ respectively. E is positive control, a treated group with 50 µM H_2_O_2_. Head DNA; amount of DNA in the comet head, Tail DNA; amount of DNA in the comet tail, Head DNA%; percentage of DNA in the comet head to comet tail, and Tail DNA%; percentage of DNA in the comet tail to comet head. Statistical analysis was calculated by One-way ANOVA test. Each point represents mean ± SD. ^a^ *P *< 0.001 compared with group A; ^b^*P *< 0.001 compared with group B; ^c^*P *< 0.001 compared with group C.

**Figure 3:**
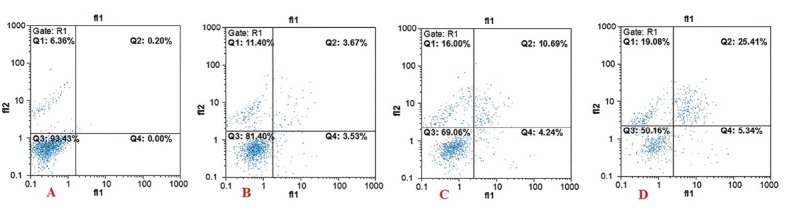
Apoptotic effects of TQ on PC3 cells after 48 h compared with control group using flowcytometry procedure. A; Negative control cells, B-D; cells treated with 20, 30 and 40 µM of TQ respectively.

DNA fragmentation is a hallmark of apoptosis that increasing *via* ROS production by TQ treated in PC3 cells.^32^ Therefore, in the present study DNA-damage can take place by ROS production which induces apoptosis. Figure 3 represents the percentage of apoptosis and necrosis after treating the cells at different concentrations of TQ (0, 20, 30, and 40 µM) related to control cells. Our results showed that the percentage of apoptosis was elevated more than that of necrosis which is in line with the previous study in GlioblastomaT-98G cells line.^33^

Also, Zhang et al showed that TQ can induce apoptosis* via* endoplasmic reticulum stress-dependent mitochondrial pathway, mitochondrial dysfunction, and activating the mitochondrion-mediated apoptotic pathway through reducing MCL-1 and MCL-XL, elevating BAX, AIF, and releasing cytochrome c.^34^ It is reported that using MD simulation studies with *Panax ginseng* derived compounds can strongly attach to three anti-apoptotic proteins including MCL-XL, MCL-1, and BCL-2 and inhibit their activities.^35^ In this study, MD simulation and docking results showed that TQ can strongly affect the MD of MCL-XL, MCL-1, and BCL-2.

Table 2 shows the binding energy (BE), final intermolecular energy (FIE), and estimated inhibition constant (EIC) of BCL-2, BCL-xl and MCL-1. According to our docking results, the lowest value of ΔG (BE) was between BCL-XL protein and TQ (Table 2). Therefore, the binding affinity of TQ to BCL-XL is higher than BCL-2 and MCL-1.

**Table 2:** Molecular interaction between TQ and BCL-2, BCL-XL, and MCL-1

**Receptor**	**BE kcal/mol**	**FIE kcal/mol**	**EIC µM**	**Interaction bonds**	
				**Hydrogen Bonding**	**Hydrophobic Bonding**
BCL-2	-4.65	-5.55	388.7	**-**	Tyr139, Arg142, His143, Glu138, Ala90, Arg86, Phe89, Trp135
BCL-XL	-5.89	-6.79	47.94	**-**	Pro116, Gly117, Ala119, Trp169, Arg165, Tyr120
MCL-1	-4.64	-5.53	397.2	**-**	Glu16, Asn223, Asn17, Gly219, Tyr21, Arg215, Val216, Tyr20

Abbreviations: BE, binding and Energy (kcal/mol); FIE, final intermolecular energy (kcal/mol); EIC, estimated inhibition constant (µM).

**Figure 4:**
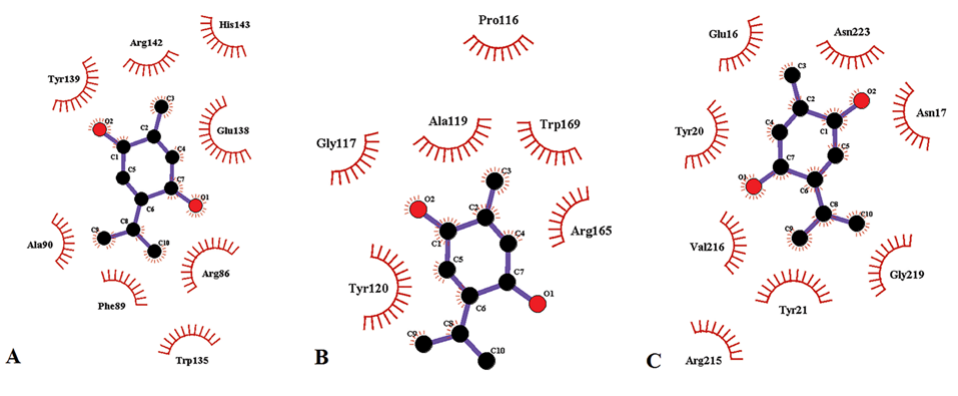
Analyzed protein–ligand interactions BCL-2 (A), BCL-XL (B), and MCL-1 (C).

The LigPlot plus results (Figure 4) showed that TQ forms eight, six, and eight hydrophobic bonds on residues of BCL-2, MCL-1, and BCL-XL, respectively. However, no hydrogen bond was found between TQ and them.

**Figure 5:**
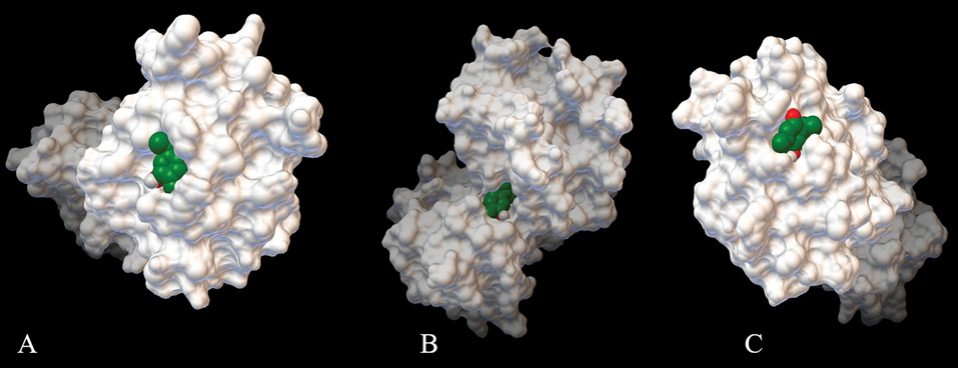
Three-dimensional structure of docked molecules between TQ and BCL-2 (A), BCL-XL (B), and MCL-1 (C).

Figure 5 shows the active sites of BCL-2, BCL-XL and MCL-1 molecules with lowest energy which are connected to TQ.

A common criterion to assess the molecular dynamic simulation and also to validate its accuracy is determining the root mean-square deviation (RMSD) value of the protein backbone compared to the initial structure during simulation. This parameter is considered as an indicator of the simulation quality and also attainment or non-attainment of the equilibrium state in the simulation system. When a simulation is running, the more RMSD for one atom or a group of atoms indicate that their structures have more variations.^36^

According to Table 3, MCL-1 molecule shows the highest variation in RMSD which has been increased in the presence of TQ. In the presence of TQ, RMSD of BCL-XL protein was reduced, subsequently, the structure changes decreased and the molecule became more stable. Therefore, it can be concluded that TQ has the more powerful inhibition effect on BCL-XL than BCL-2 and MCL-1.

**Table 3:** Molecular dynamic parameters

**Protein**		**Rg**	**RMSD**	**RMSF**
Bcl-2	G_1_	2.22±0.020	0.58±0.112	0.190±0.061
	G_2_	2.21±0.033^a^	0.25±0.057^a^	0.250±0.080^a^
Bcl-xl	G_1_	1.96±0.016	0.68±0.043	0.205±0.072
	G_2_	1.40±0.005^a^	0.32±0.010^a^	0.155±0.080^a^
Mcl-1	G_1_	2.89±0.023	0.27±0.04	0.160±0.056
	G_2_	2.48±0.027^a^	0.68±0.15^a^	0.330±0.120^a^

Abbreviations: G_1_, simulation before docking; G_2_, simulation after docking; Rg, Radius of gyration; RMSF, root mean square fluctuation; RMSD, root mean-square deviation. Statistical analysis was calculated by paired-sample *t* test. Each point represents mean ± SD. ^a^ *P*< 0.001 compared with group G_1_.

To assess the oscillation of each residue, we measured root mean square fluctuation (RMSF) to observe the flexibility of key residues. RMSF values in the presence of TQ for the BCL-2 and MCL-1 increased but for BCL-XL decreased (Table 3).

**Figure 6:**
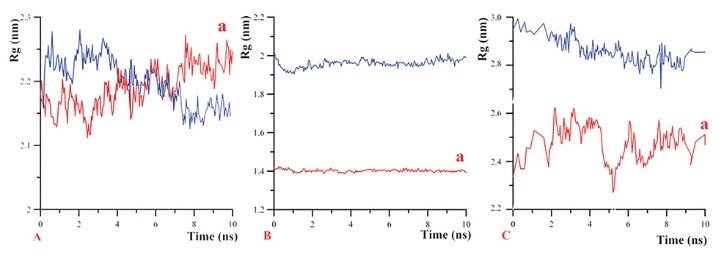
Radius of gyration (Rg) for BCL-2 (A), BCL-XL (B) and MCL-1 (C). Blue color; simulated anti-apoptotic factors in the absence of TQ and red color; simulated anti-apoptotic factors in the presence of TQ. Statistical analysis was calculated by Paired-sample *t* test. Each point represents mean±SD. ^a^*P* < 0.001 compared with Blue color.

Radius of gyration (Rg) is a scale of the protein radius. For globular proteins, the more Rg means the less compaction of the protein structure and more function. In the presence of an effective drug, Rg of the protein changes and subsequently its structure is altered.^37^ This study showed that, TQ could decrease the Rg properties of these anti-apoptotic factors significantly. But, the most effect of TQ was on BCL-XL and MCL-1 (Figure 6 and Table 3).

The variations in the secondary structures of MCL-1, BCL-XL, and BCL-2 and the values of these variations as a single molecule or in complex with TQ are displayed in Table 4. According to the variation in the secondary structure parameter of the studied proteins, it seems that when TQ binds to BCL-XL, alpha-helix structure of the protein decreases and Bend, Turn and Coil structures decrease significantly (Table 4). Since alpha helix and beta-sheet structures exist in structural regions of proteins and turn and coil structures mostly exist in functional regions.^38^ It is hypothesized that in this state, BCL-XL is less effective. MCL-1 and BCL-2 molecule displayed fewer variations than BCL-XL. However, some of these variations are significant but the biggest 2D structure variation is related to BCL-XL.

**Table 4:** The variations in secondary structure of MCL-1, BCL-XL and BCL

**Protein**		**Coil**	**Bend**	**Turn**	**α-Helix**
Bcl-2	G_1_	41.87±2.09	14.10±3.20	22.45±4.06	201.86±3.88
	G_2_	42.13±2.18^a^	16.95±2.94^a^	21.31±4.02^a^	201.70±3.87
Bcl-xl	G_1_	38.96±1.7	13.21±3.20	24.87±4.80	199.56±6.03
	G_2_	19.45±1.7^a^	7.45±2.41^a^	11.62±3.65^a^	194.92±4.16^a^
Mcl-1	G_1_	47.36±2.07	18.42±2.90	22.58±3.75	244.76±3.17
	G_2_	47.47±2.45	22.22±4.45^a^	23.75±4.76^a^	239.90±4.18^a^

Abbreviations: G_1_, simulation before docking; G_2_, simulation after docking. Statistical analysis was calculated by paired-sample *t* test. Each point represents mean±SD. ^a^ *P* < 0.001 compared with group G_1_.

## Conclusion

This study showed that TQ at the concentrations close to IC_50_ can damage PC3 PC cells and also induce apoptosis more than necrosis. Molecular docking results and analysis revealed that TQ makes more stable bonds with BCL-XL than BCL-2 and MCL-1. RMSD data indicated that TQ inhibits BCL-XL non-competitively, so by inhibiting BCL-XL and less MCL-1, TQ can induce apoptosis. MD results indicated that TQ has the lowest influence on MCL-1. The changes in the secondary structure showed that TQ exerts drastic changes over BCL-Xl molecule by reducing the effective 2D structures. Taken together, our study revealed that TQ induces apoptotic effects by inhibiting the anti-apoptotic factor BCL-XL protein compared to other studied factors.

## Ethical Issues

The current article does not contain any studies with human or animal subjects.

## Conflict of Interest

The authors declare that there is no conflict of interest.

## Acknowledgments

This paper was based on a research project (no: 1682) funded by deputy of research and technology of Shahrekord university of medical sciences, Shahrekord, Iran. We gratefully thank this deputy.
